# The Degree of Inulin Polymerization Is Important for Short-Term Amelioration of High-Fat Diet (HFD)-Induced Metabolic Dysfunction and Gut Microbiota Dysbiosis in Rats

**DOI:** 10.3390/foods13071039

**Published:** 2024-03-28

**Authors:** Amin Ariaee, Hannah R. Wardill, Anthony Wignall, Clive A. Prestidge, Paul Joyce

**Affiliations:** 1 UniSA Clinical and Health Sciences, University of South Australia, Adelaide, SA 5000, Australia; amin.ariaee@mymail.unisa.edu.au (A.A.); anthony.wignall@unisa.edu.au (A.W.); clive.prestidge@unisa.edu.au (C.A.P.); 2 School of Biomedicine, The University of Adelaide, Adelaide, SA 5000, Australia; hannah.wardill@adelaide.edu.au; 3 Supportive Oncology Research Group, Precision Cancer Medicine, South Australian Health and Medical Research Institute, Adelaide, SA 5000, Australia

**Keywords:** gut microbiome, dysbiosis, obesity, metabolic disease, prebiotics, inulin, fructooligosaccharides

## Abstract

Inulin, a non-digestible polysaccharide, has gained attention for its prebiotic properties, particularly in the context of obesity, a condition increasingly understood as a systemic inflammatory state linked to gut microbiota composition. This study investigates the short-term protective effects of inulin with different degrees of polymerization (DPn) against metabolic health deterioration and gut microbiota alterations induced by a high-fat diet (HFD) in Sprague Dawley rats. Inulin treatments with an average DPn of 7, 14, and 27 were administered at 1 g/kg of bodyweight to HFD-fed rats over 21 days. Body weight, systemic glucose levels, and proinflammatory markers were measured to assess metabolic health. Gut microbiota composition was analyzed through 16S rRNA gene sequencing. The results showed that inulin_27_ significantly reduced total weight gain and systemic glucose levels, suggesting a DPn-specific effect on metabolic health. The study also observed shifts in gut microbial populations, with inulin_7_ promoting several beneficial taxa from the *Bifidobacterium* genera, whilst inducing a unique microbial composition compared to medium-chain (DPn 14) and long-chain inulin (DPn: 27). However, the impact of inulin on proinflammatory markers and lipid metabolism parameters was not statistically significant, possibly due to the short study duration. Inulin with a higher DPn has a more pronounced effect on mitigating HFD-induced metabolic health deterioration, whilst inulin_7_ is particularly effective at inducing healthy microbial shifts. These findings highlight the benefits of inulin as a dietary adjuvant in obesity management and the importance of DPn in optimizing performance.

## 1. Introduction

Obesity has emerged as a global epidemic, affecting millions of adults and imposing a substantial economic burden estimated to be at least USD 2.0 trillion [1]. Current interventions, including pharmacotherapies and lifestyle modifications, are often met with limited success, attributed largely to patient non-adherence [2]. This is partly attributed to the adverse events associated with the pharmacotherapies, including neuropsychiatric and cardiovascular complications, compromising their efficacy and patient compliance [3,4]. 

Recent advancements in our understanding of obesity have shifted away from the historical perspective of an energy imbalance between caloric intake and expenditure towards the concept that obesity is in fact a systemic inflammatory condition [5,6]. The initiation of inflammation in obesity is complex and multifaceted; genetic susceptibility, environmental influences, and significantly, the composition and function of the gut microbiome all contribute to its prevalence [7,8,9]. The gut microbiome, with its vast array of around 40 trillion bacteria, is often perturbed in obesity, leading to an increase in harmful pathobiont species at the expense of beneficial commensal bacteria, commonly known as gut dysbiosis [10]. Key factors in the development of dysbiosis include dietary habits, particularly the lack of microbial-accessible carbohydrates that are crucial for a healthy microbiome [11,12]. 

Inulin, a naturally occurring polysaccharide, has been the subject of extensive research in obesity and other metabolic diseases [13,14,15]. Characterized by its β(2→1) fructosyl–fructose glycosidic bonds, inulin evades host digestion and is metabolized by selective microbiota species producing inulinase enzymes [16]. This substrate-specific interaction acts as an energy source for the microbiota but also instigates the production of metabolites such as short-chain fatty acids (SCFAs), which mitigate inflammatory pathways through mechanisms such as the modulation of G-protein-coupled receptors (GPCRs) and toll-like receptors (TLRs) as well as the enhancement of intestinal barrier integrity [17,18,19]. As one of the major SCFA components, butyrate is known to enhance the integrity of the intestinal barrier by promoting the assembly of tight junction occludin and claudin-2 proteins, thereby reducing the translocation of pro-inflammatory bacterial components such as lipopolysaccharides (LPSs) from the gut lumen into the bloodstream [20]. 

SCFAs that act as ligands for GPCRs, such as GPR41 and GPR43, inhibit nuclear factor-kappa B (NF-κB) pathways, leading to the decreased production of pro-inflammatory cytokines, such as tumor necrosis factor-alpha (TNF-α) and interleukin-6 (IL-6) [21]. Additionally, inulin’s impact on the gut microbiota composition itself can influence inflammatory pathways. By promoting the growth of beneficial commensal bacteria, such as *Bifidobacterium*, inulin may outcompete potentially harmful pathobiont bacteria for ecological niches, reducing their ability to induce inflammation [22,23]. These beneficial microbes not only produce SCFAs but can also directly modulate the immune system, further contributing to an anti-inflammatory gut environment [24]. Although the beneficial impacts of inulin are well documented across various disease models, including obesity, the impact of inulin’s degree of polymerization (DPn) on metabolic health and the gut microbiota is not well established. Recent studies have demonstrated the benefits of long-chain inulin (DP ≥ 22) for reducing weight gain in small animal models of obesity; however, these studies are limited by their small range and poor characterization of inulin DPn [25,26]. Moreover, these studies have focused on the impacts of inulin for longer treatment durations of between 8 and 10 weeks, but little is known about their effects for shorter treatment periods. Hence, this study aims to explore both the metabolic and microbial impacts of inulin, with 7, 14, and 27 DPn, on a Sprague Dawley rat model of obesity over a 3-week study period.

## 2. Materials and Methods

### 2.1. Materials

Inulin from chicory root was sourced from Pharmako Biotechnologies (Frenchs Forrest, Australia). Inulin sourced from chicory, inulin from Dahlia tubers, phosphate buffered saline (PBS) tablets, 2-ethyl butyrate, analytical-grade acetic acid, butyric acid, and propionic acid were purchased from Sigma-Aldrich (Castle-Hill, Australia). Five-week-old Sprague Dawley rats were obtained from the Animal Resource Centre (Perth, Australia). Ultra-pure Milli-Q water was used throughout all studies. 

### 2.2. In Vivo Study Design 

The in vivo study was approved by the Animal Ethics Committee of the University of South Australia (approval #24-21), strictly following the Principles of Laboratory Animal Care (NIH publication #85-23) and reported according to ARRIVE guidelines. The HFD-induced rodent study was adapted from previous obesity models [27,28]. The study was statistically powered according to predicted changes in bodyweight to a power level of more than 0.8. Five-week-old Sprague Dawley rats were placed in groups of three in separate cages. After a week of acclimatization, rats were separated into the following groups (*n* = 6): HFD (44% kcal from dietary fats), HFD + inulin_7_ (average DPn of 7), HFD + inulin_14_, and HFD + inulin_27_ (inulin oral gavage at 1 g/kg bodyweight/d). Inulin was dispersed daily in fresh phosphate buffer saline (10 mL/kg) prior to its administration between 16:00 and 17:00 for 21 days. Daily bodyweight measurements were taken prior to dosing. 

### 2.3. Post In Vivo Study Sample Collection and Analysis

Following the final dose at 21 d, rats fasted for 24 h prior to being anaesthetized with illium sodium pentobarbital (60 mg/mL) at 200 mg/kg bodyweight. Approximately 3 mL blood samples were collected via a cardiac puncture and analyzed by SA Pathology (Adelaide, Australia). After humane culling, the liver, cecum, and fat pads (retroperitoneal, epididymal, and mesenteric) were dissected. Equal parts fecal and cecal samples were combined and sent to for DNA extraction and 16S rRNA sequencing at the Australian Genomics Research Facility (Brisbane, Australia). Hypervariable V3-V4 regions were selected, and the raw output was clustered based on 97% similarity to operational taxonomic units (OTUs) using the Quantitative Insights into Microbiology Ecology (QIIME 2) and the Silva reference database [29,30]. Taxonomy was assigned to OTUs using Qiagen (Hilden, Germany) CLC Genomics Workbench Version 23.0.4 and its Microbial Insights—Prokaryotic Taxonomy Database (QMI-PTDB) [31]. Alpha diversity plots were based on the Shannon Index, and beta diversity Principal Coordinate Analyses (PCoAs) were derived from Bray–Curtis dissimilarity and Jaccard units [32,33]. To investigate the genetic similarity and diversity within 16s data, a hierarchical clustering of k-mers was also performed using a fixed k-mer length of 16 nucleotides. Statistical significant differences in beta diversity were calculated using permutational multivariate ANOVA (PERMANOVA) [34]. To visualize the variation in and clustering of microbial communities associated with different treatments, the samples were grouped on the PCoA plot using a 95% confidence ellipse. 

### 2.4. Serum Proinflammatory and Lipid Analysis Using ELISA

Serum was separated from clotted whole blood via centrifugation (2000× *g*, 20 min, 4 °C) and collection of the supernatant. Interleukin (IL)-6 and TNF- α were quantified from rat serum using ELISA kits (ThermoFisher Scientific, Adelaide, Australia), according to the manufacturer’s manual. *n* = 4–6 was used for all ELISA kits due to outliers in the data. 

### 2.5. In Vitro Intestinal Cell Permeability Studies Using SCFAs

Caco-2 cells were seeded on 12-well permeable inserts at a density of 2.5 × 10^5^ cells mL^−1^. Cells were kept incubated in 5% CO_2_ until full differentiated (21 d) to represent a functional human intestinal epithelial monolayer. Cell media in the wells were replaced every 2 d. TEER was measured using a Millicell-ERS volt-ohmmeter (Millipore, Bedford, NY, USA) both before and after treatment with LPS and/or SCFAs at different concentrations and time points. Electrodes were placed on both the basolateral and apical compartments of the inserts and resistance was measured across the Caco-2 cell monolayer. Wells were measured repeatedly until the same value (±50 Ω cm^2^) was observed on 3 consecutive attempts. Only cell monolayers with a TEER above 400 Ω cm^2^ were used for treatment. Recovery of the monolayer was expressed as a percentage of its starting TEER value prior to treatment. All experimental groups were *n* = 3. FITC-dextran was used as a paracellular marker for the Caco-2 cell monolayer model. Apical cell media were removed from the treatment groups and replaced with DMEM (0.5 mL) containing 150 μg mL^−1^ FITC-dextran (4 kDa). After 20 min of incubation under 5% CO_2_ conditions at 37 °C, 100 uL of the basolateral media was collected and analyzed using a microplate reader (Perkin Elmer Victor3 1420 Multilabel Counter, Waltham, MA, USA) at excitation wavelength of 485 nm and emission wavelength of 535 nm. Permeability was expressed as a percentage of the negative control group.

### 2.6. In Vitro Intestinal Cell Permeability Studies Using CFS

Commensal *Blautia* strain was incubated at 37 °C on yeast, casitone, and fatty acid (YCFA) media, which are selective for the cultivation of anaerobic bacteria, using a previously described method [35]. Briefly, Blautia strains were grown at an optical density of ~600 nm co-dosed with inulin_14_ overnight. The culture’s cell-free supernatant (CFS) was isolated by centrifugation (5000× *g*, 10 min, 4 °C) and filtering (0.2 μm Millipore filter). 

### 2.7. Fecal and Cecal Short-Chain Fatty Acid Quantification

SCFA concentrations of acetic, butyric, and propionic acids were analyzed using Shimadzu gas chromatography–mass spectrometry ((GCMS)-QP2010 SE (Kyoto, Japan)), following the parameters set out in a previous protocol [36]. 

### 2.8. Statistical Analysis

Experimental data (excluding 16S rRNA gene sequencing) were statistically analyzed using GraphPad Prism Version 8.0 (Boston, MA, USA). Statistically significant differences were deduced using one-way and two-way ANOVA, with multiple comparisons conducted using Tukey’s post hoc test. Reported values are represented as mean ± standard deviation (SD), and statistical significance is assumed when *p* < 0.05. Statistical significance is annotated as * *p* < 0.05, ** *p* ≤ 0.01, *** *p* ≤ 0.001, or **** *p* ≤ 0.0001.

## 3. Results and Discussion

### 3.1. Impact of Inulin’s DPn on Diet-Indued Metabolic Health of Sprague Dawley Rats

**Figure 1:**
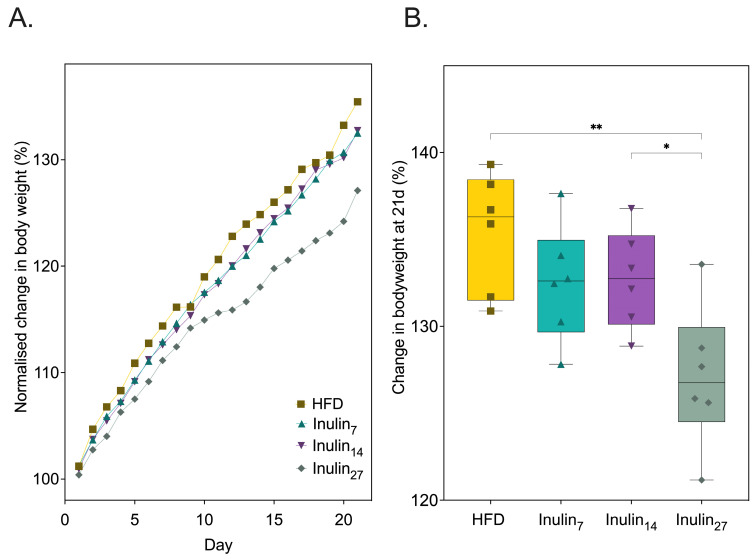
(**A**) Normalized change in rodent bodyweight over 21 days of HFD and treatment with inulin of various DPn. (**B**) The total bodyweight gain at day 21 was significantly reduced for inulin_27_ compared to all other groups. Statistical significance is annotated as * *p* < 0.05, and ** *p* ≤ 0.01.

To investigate the impact of inulin’s DPn on health metabolic markers, HFD-fed rats were administered with inulin daily with an average DPn of 7 (i.e., fructooligosaccharides), 14, and 27 over 21 days. Figure 1 shows that at the end of the treatment phase, only inulin_27_ significantly reduced HFD-diet-induced total weight gain by 6.1% (*p* = 0.0025). This finding supports previous studies that validated the weight loss effects of inulin supplementation in a small animal model of obesity. The predominate hypothesis behind inulin’s weight loss effect is its modulation of the gut microbiota—promoting beneficial commensal taxa such as *Bifidobacterium* and Lactobacillus [26,37,38]. Inulin fermentation by the microbiota produces SCFA metabolites with the major products being acetic acid, propionic acid, butyric acid, and their conjugate bases [39,40]. The SCFA can reduce weight gain via regulating the secretion of appetite-controlling hormones, such as glucagon-like-peptide 1 (GLP-1) and Peptide YY (PYY), in order to increase satiety [41]. Moreover, SCFAs regulate lipid metabolism by stimulating fatty acid oxidation and mitochondrial activity through adenosine monophosphate-activated protein kinase (AMPK) signaling and uncoupling protein (UCP) expression, while also affecting lipid synthesis and storage by acting as substrates and modulating key enzymes and hormonal factors, e.g., hormone-sensitive lipase (HSL), adipose triglyceride lipase (ATGL), and leptin [42,43,44]. 

Inulin_27_ administration caused a significantly (*p* < 0.05) lower weight gain of 127 ± 3.74 g compared to 132 ± 3.05 g and 133 ± 2.61 g for inulin_7_ and inulin_14_, respectively (Figure 1B). This is consistent with other HFD rodent studies demonstrating the pronounced effects of longer-chain (DPn < 22) inulin for reducing diet-induced weight gain when compared to short-chain inulin (DPn 2-8) [25,26]. These studies showed that long-chain inulin significantly impeded the levels of lipid-metabolism-associated genes, acetyl-CoA carboxylases (ACC), sterol regulatory element-binding protein 1 (SREBP1), and fatty acid synthase (FAS) expression in the liver of HFD-fed mice. Hence, these results suggest a higher inulin DPn downregulates lipogenesis pathways. 

### 3.2. Metabolic and Inflammatory Biomarker Responses to Inulin Supplementation

**Figure 2:**
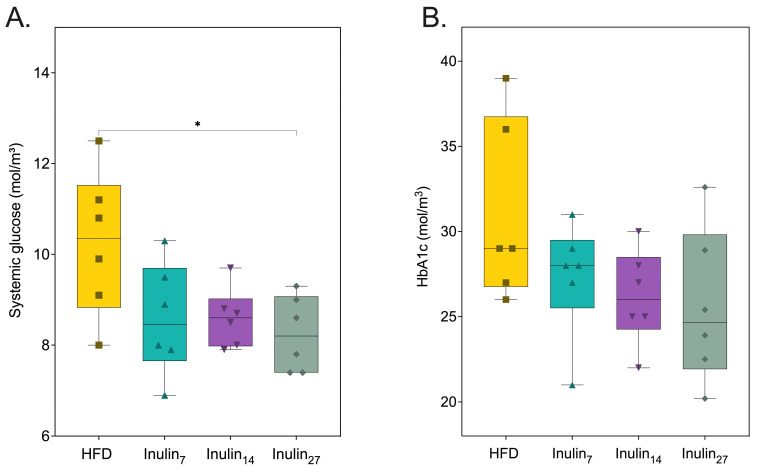
(**A**) Systemic glucose was reduced by inulin27, whilst (**B**) glycated hemoglobin A1C levels remained the same for all groups at 21 days of inulin treatment for HFD-fed rats. Statistical significance is annotated as * *p* < 0.05.

Systemic glucose and glycosylated hemoglobin (HbA1c), reflecting the average glucose levels over a 2–3 month period, were measured and are displayed in Figure 2 [45]. The rats supplemented with inulin_27_ had a 19% (*p* = 0.0298) reduction in their systemic glucose levels, compared to those of the HFD control. No other inulin groups had statistically significantly decreased systemic glucose levels after the 21-day treatment period, but a clear trend highlighting the capacity for each inulin type to reduce blood glycemic levels was observed. While HbA1c levels decreased proportionally with increases in inulin DPn, the changes were not statistically significant, possibly due to the limitations posed by the statistical approaches utilized and the short duration of the study. This view is supported by a study conducted by Miralles-Pérez et al., demonstrating that inulin (15% *w*/*w* of diet) reduced HbA1c levels in HFD-rats after 10 weeks [46]. 

**Figure 3:**
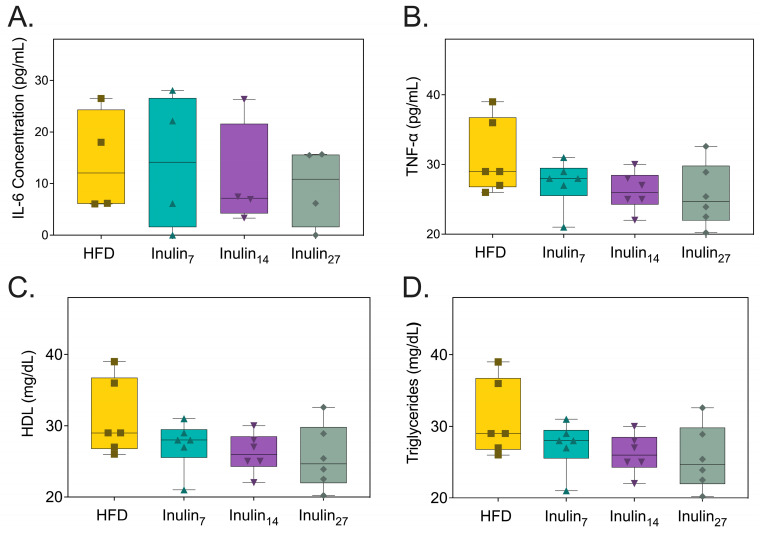
Metabolic markers of health measured from rats after 21 days of treatment. (**A**) Serum IL-6 concentration, (**B**) TNF-α concentration, (**C**) HDL concentration, and (**D**) triglyceride concentrations were not affected by inulin treatments.

With obesity being increasingly recognized as a condition of systemic inflammation, proinflammatory markers associated with the disease were measured after the inulin treatment. Figure 3A shows the serum Interleukin (IL)-6 concentrations at the end of the treatment phase were 28% for the inulin_7_ and inulin_27_ groups and 40% for inulin_14_ when compared to the HFD group (not significant). Similarly, the TNF-α levels for the HFD group were reduced by 10% and 6.0% for inulin_14_ and inulin_27_, whilst those of inulin_7_ increased by 8.8% (not significant). Lipid metabolic parameters, systemic triglycerides, and high-density lipoprotein (HDL) levels were also unchanged by the administration of inulin for 21 days (Figure 3C,D). Although there appears to be greater anti-inflammatory effects with the administration of inulin_14_, the lack of statistically significant differences between the groups may be due to the shorter 21-day duration of the current study. This was also observed when Komatsu et al. reported that 28 days of a high-inulin diet (20% *w*/*w*) did not induce statistically significant changes to systemic IL-6, triglyceride, and HDL levels compared to control diet (5% *w*/*w*) in DahlS.Z-*Lepr^fa^/Lepr*^fa^ (DS/obese) rats [47]. However, a longer 16-week study on HFD-fed (60% of their diet in terms of calories) C57/BL mice supplemented with inulin (10% *w*/*w*) found significantly (*p* < 0.05) lower serum triglyceride, TNF-a, and Il-6 levels, whilst HDL levels remained unchanged [48]. An HFD has been linked to inflammation via a phenomenon termed ‘metabolic endotoxemia’ [49]. High-fat diets in mice increase the plasma concentration of lipopolysaccharide (LPS), a bacterial endotoxin that is the marker characteristic of low-grade systemic inflammation [50]. LPS is a major component of the outer membrane of Gram-negative bacteria, known to elicit a robust immune response when exposed to the intestinal epithelium [51,52]. One study of Gambian women found that obesity-related diabetic patients have 57% higher serum LPS levels than healthy individuals [53]. Systemic LPS contributes to metabolic endotoxemia in obesity by activating toll-like receptor 4 on innate immune cells such as macrophages, triggering two pathways: the MyD88-dependent route initiating proinflammatory cytokine production; and the MyD88-independent pathway, which promotes interferon responses. Both lead to a complex inflammatory state that exacerbates obesity via the activation of NF-κB and p38 mitogen-activated protein kinase (MAPK) pathways [54,55,56,57,58]. 

### 3.3. DPn of Inulin Influencing the Gut Microbiome

**Figure 4:**
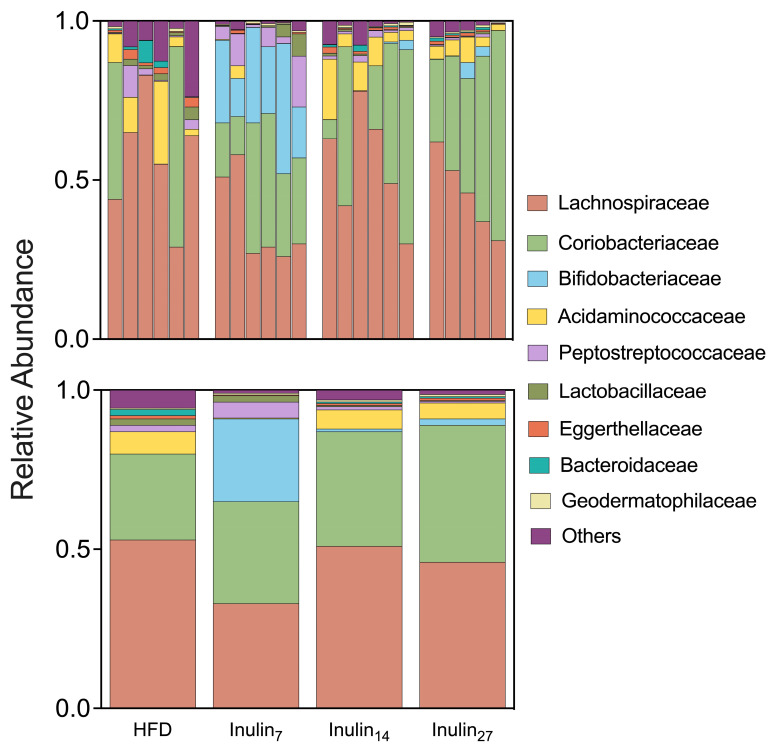
The relative abundance taxa at the family level are shown for each rat sample (**top**) and the combined treatment groups (**bottom**). Inulin_7_ showed lower abundance of *Lachnospiraceae* associated with metabolic disease, whilst promoting *Bifidobacteriaceae* taxa containing several beneficial commensal species.

Our study aimed to investigate the impact of inulin DPn on the microbiome induced by a HFD in rats over a 21-day period. Utilizing 16S rRNA gene sequencing data from fecal and cecal samples, distinct shifts in microbial populations were observed with implications for gut health and metabolic processes. An analysis of the relative abundance of microbial taxa at the family level revealed significant alterations in the gut microbiome composition across different treatment groups (Figure 4). The HFD group showed a 37% higher relative abundance of *Lachnospiraceae* compared to the inulin_7_-treated rats, consistent with previous findings in germ-free obese mice colonized with *Lachnospiraceae* bacteria (strain AJ110941), which showed elevated amounts of liver and mesenteric adipose tissue [59]. Similarly, supplementation with inulin_7_ led to a 11 log_2_-fold increase in the relative abundance of Bifidobacteriaceae, a taxon containing species such as Bifidobacterium longum APC1472 that are associated with anti-obesity effects in humans [60]. These changes reflect the preferential prebiotic effects of inulin_7_ compared with longer-chain inulin in diet-induced obese rats. 

**Figure 5:**
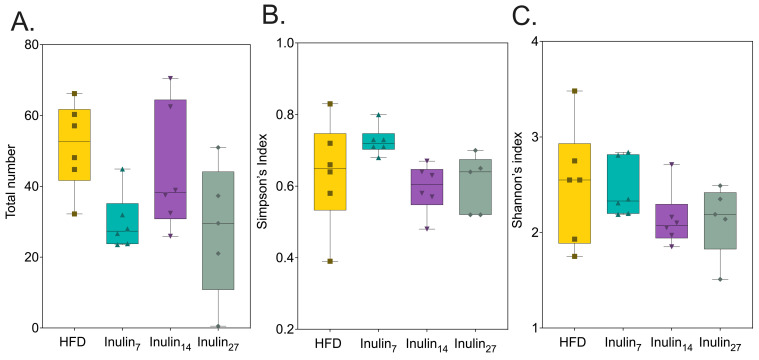
Alpha-diversity presented as box-and-whisker plots illustrating no statistical differences amongst inulin treatments and the HFD group in terms of (**A**) the total number of species or OTUs across treatments, (**B**) Simpson’s index (accounting for the number of species and the abundance of each species), and (**C**) Shannon index (considering both the abundance and evenness of the species present).

The alpha-diversity indices exhibited no significant differences in the microbial diversity between the inulin and HFD treatment groups (Figure 5). This finding is in contrast to that of a previous study demonstrating short-chain inulin (DP 4–5) but not long-chain inulin (DP 23–25) increased the Chao1-measured alpha-diversity in HFD-fed mice after 10 weeks [25]. This discrepancy in our findings might be attributed to the duration of the inulin treatments, as the prebiotic effects of inulin on microbial communities have been more consistently observed over longer periods, ranging from 6 to 17 weeks [61,62,63]. Additionally, the differences could also be due to variations in study power, with the previous studies using between eight and twelve animals per group, influencing the sensitivity to detect changes in microbial diversity.

**Figure 6:**
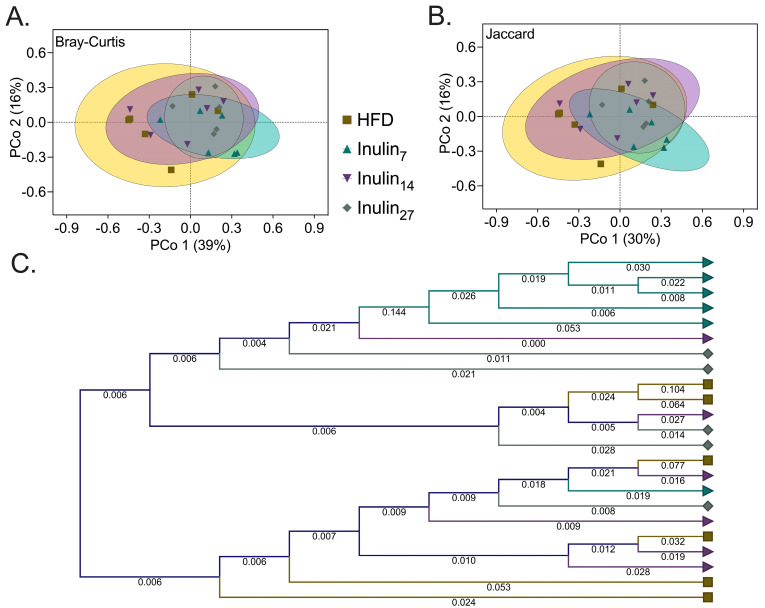
Beta-diversity is presented as PCoA plots, with each illustrating differences between inulin_7_ and all other groups using (**A**) Bray–Curtis dissimilarity (representing the differences in species composition between samples); and (**B**) Jaccard index (the presence and absence of species used to visualize the dissimilarity between the samples). (**C**) Hierarchical k-mer clustering (k-mer length = 16) represents the phylogenetic distance between the HFD (■), inulin_7_ (▲), inulin_14_ (▲) and inulin_27_ (◆) microbial compositions.

Beta-diversity measurements were also generated as PCoA plots to identify the degree of similarity/dissimilarity in the gut microbiome community between treatment groups (Figure 6A,B). The Bray–Curtis and Jaccard indices demonstrate the substantial differences in microbial composition for the inulin_7_ treatment compared to all other groups (Table 1, *p* < 0.05). Similarly, the hierarchical clustering of the k-mer profiles demonstrate that inulin_7_ has the highest divergence in its genetic composition of the microbiome compared to the HFD group, as shown by its longer branch lengths in those samples (Figure 6C). Differences in the microbial communities promoted by inulin_7_ compared to the longer-chain inulin were suggested by Li et al. to be due to the more selective utilization of complex polysaccharides (DP > 7) by species, leading to unique microbial compositions depending on the inulin chain length [25]. This view is supported by another study that found short-chain inulin (DP < 10) increased in vitro batch fermentation (human fecal inoculum) by 58% more than long-chain inulin (DP > 20) after 4 h [64]. These studies indicate that short-chain and long-chain inulin can modulate the gut microbiome; specific microbial compositions differ based on how rapidly inulin can be utilized by a species. 

**Figure 7:**
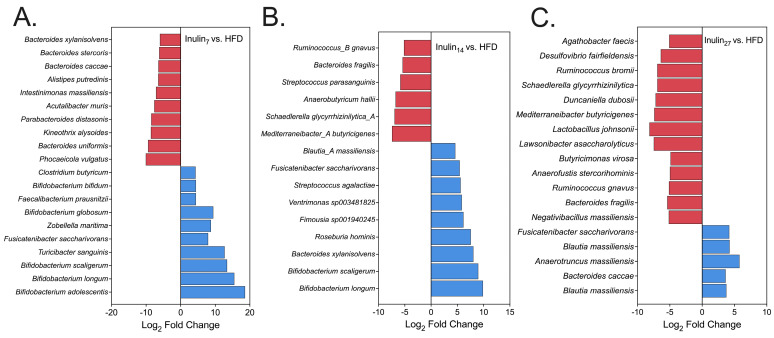
Differential abundance analysis showing statistically significant differences (*p* < 0.05) in the total abundance of all species for rats that were treated with either (**A**) inulin_7_, (**B**) inulin_14_, or (**C**) inulin_27_ compared to the HFD group. Species depicted in red have been reduced, and blue have been promote, compared to the HFD group.

A differential abundance analysis between the microbial species unveiled statistically significant shifts (*p* < 0.05) in several key bacteria associated with health and obesity (Figure 7). The inulin_7_ and inulin_14_ treatments had potent bifidogenic effects, increasing between 4.4 and 18 log_2_ fold when compared to the HFD group. Species from the Blautia genera were also promoted by both inulin_14_ and inulin_27_, with Blautia depletion having previously been associated with visceral fat accumulation and insulin resistance in humans [65,66]. Another key commensal species, *Faecalibacterium praustnitzii*, was enhanced 4.4 log_2_ fold by inulin_7_. This strain led to a lower hepatic fat content and increased fatty acid oxidation in HFD-fed mice after 2 weeks of intragastric *F. praustnitzii* (2 × 10^8^ CFU) inoculation [67]. Additionally, Yang et al. found that *F. prausnitzii* strains (1 × 10^8^ CFU) inoculated in HFD-induced mice for 12 weeks reduced weight gain by regulating the expression of lipid-metabolism-associated genes, such as leptin, FAS, SREBP1c, and adiponectin [68]. Hence, the inulin_7_ treatment significantly altered the gut microbiome in HFD-fed rats, enhancing several beneficial species linked to reduced obesity and improved metabolic outcomes.

### 3.4. SCFA Recovery of In Vitro Intestinal Epithelial Permeability

**Figure 8:**
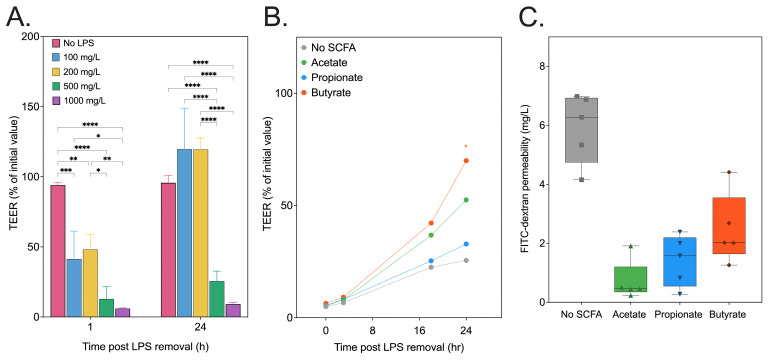
(**A**) Transepithelial electrical resistance (TEER) of Caco-2 cell monolayers measured at 1 h and 24 h following LPS (0–1000 mg/L) removal from the culture medium. Concentrations of 500 and 1000 mg/L of LPS are sufficient to induce sustained Caco-2 monolayer layer after 24 h. (**B**) The recovery of TEER when SCFAs are added to the culture medium at 10 mol/m³ following LPS (500 mg/L) exposure at 0 h, 3 h, 18 h, and 24 h reveals only butyrate restores TEER, whilst all treatments show decreased (**C**) FITC-dextran permeability after LPS exposure. TEER was measured at each timepoint as the % of Transwell initial TEER measurement prior to the start of treatment. Statistical significance is annotated as * *p* < 0.05, ** *p* ≤ 0.01, *** *p* ≤ 0.001, or **** *p* ≤ 0.0001.

The underlying mechanisms of inulin’s benefits for metabolic health were explored further in vitro. Specifically, we investigated SCFAs, known metabolites produced by the fermentation of inulin by the gut microbiome, and their contribution to intestinal barrier integrity. Using a well-established in vitro model, we examined the role of SCFAs in reversing lipopolysaccharide (LPS)-induced damage to Caco-2 cell monolayers, a marker for gut epithelial permeability [69]. SCFAs reduce inflammation in obesity by binding to G-protein coupled receptors (GPCRs) on intestinal cells, leading to the enhancement of gut barrier function against inflammatory agents, and act as histone deacetylase inhibitors to modulate gene expression related to inflammation. Hence, we studied the impact of these SCFAs on intestinal epithelium permeability post LPS-induced damage. Concentration-dependent permeability assays were conducted on immortalized colorectal adenocarcinoma (Caco-2) cells using LPS between 0 and 1000 mg/L. This study was conducted to find an appropriate LPS concentration for the in vitro model whereby the dysfunction in monolayer permeability is sufficiently sustained up to 24 h post LPS removal from the cell growth medium. Transepithelial electrical resistance (TEER) across the Caco-2 monolayer was measured 1 h, 2 h, and 24 h after the removal of the medium with LPS (Figure 8A). An hour after the LPS removal, a positive correlation existed between permeability damage resulting from higher LPS concentration and TEER, which decreased between 56% (100 mg/L) and 94% (1000 mg/L) compared to the non-LPS control medium. However, whilst both the 100 mg/L and 200 mg/L groups fully recovered, the 500 mg/L and 1000 mg/L LPS groups had sustained reductions of 75% and 91%, respectively (*p* < 0.0005). Hence, the former LPS concentration was selected to induce Caco-2 permeability damage, with the SCFAs dosed post LPS removal to assess their impact on the recovery of permeability function (Figure 8B). TEER recovery was only observed for butyrate at 24 h post LPS removal from the cell growth medium (*p* < 0.05). The permeability of fluorescein isothiocyanate (FITC)–dextran was also assessed across the Caco-2 cell monolayer (Figure 8C). After 24 hours of LPS exposure, all SCFAs had significantly decreased levels of LPS-induced permeability (between 64% and 88% (*p* < 0.0005)). These findings are consistent with those of a previous study exhibiting the recovery of TEER and the attenuation of permeability marker transport during in vitro Caco-2 cell studies by SCFAs [70]. The benefits of butyrate for Caco-2 barrier function are likely due to its enhancement of tight junction proteins, which regulates paracellular transport across the intestinal epithelium [71]. As inulin fermentation by the gut microbiota produces SCFAs, it is likely their role in the function of the gut barrier reduces the low-grade systemic inflammation levels observed in obese subjects.

### 3.5. Short-Chain Fatty Acid Metabolites Following Inulin Administration to HFD-Fed Rats

**Figure 9:**
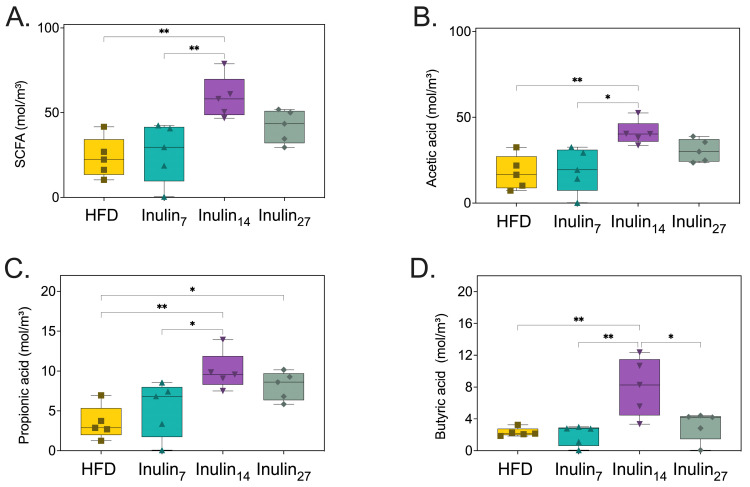
Cecal and fecal concentrations of (**A**) total cumulative SCFAs content, (**B**) acetic acid, (**C**) propionic acid, and (**D**) butyric acid are increased by inulin_14_ administration to HFD-fed rats. Statistical significance is annotated as * *p* < 0.05, and ** *p* ≤ 0.01.

Following 21 days of treatment to the HFD-fed rats, cecal and fecal samples were collected and analyzed for their content of major SCFA products. Inulin_14_ administration enhanced HFD-rat SCFA concentration by 2.5 fold (*p* < 0.005), whilst inulin_7_ and inulin_27_ had no statistically significant impact on SCFA levels (Figure 9A). When analyzing the major SCFA products, only inulin_14_ significantly increased acetate, butyrate, and propionate concentrations between 2.3 and 3.5 fold (*p* < 0.005), and inulin_27_ also increased the concentration of the latter by 2.3 fold (*p* < 0.05) (Figure 9B–D). It has been established that the DPn of inulin significantly affects its fermentability, with simpler β-fructans structures such as FOS exhibiting a 50% higher fermentation rate than inulin during in vitro fermentation studies [72]. However, the trend is not as clear for the SCFA profiles of each inulin type, with Stewart et. al. demonstrating that a combination of longer- and short-chain inulin produced significantly higher total SCFAs and acetate in an in vitro fermentation model [64]. This supports our current findings and may be due to the bacterial species’s preferential utilization of inulin with varying degrees of polymerization [73]. A combination of short- and long-chain inulin allows for a wider spectrum of bacterial species to ferment the prebiotic, resulting in a higher amount of metabolite (i.e., SCFA) production. 

### 3.6. Inulin-Derived Metabolites Protecting against In Vitro LPS-Induced Intestinal Epithelial Permeability

**Figure 10:**
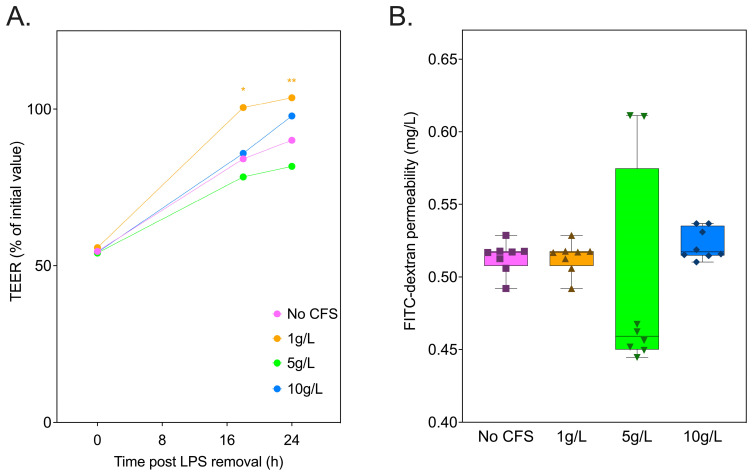
Cell-free supernatant (CFS) at 1 g/L of inulin_14_ fermented by commensal *Blautia* strains recovered TEER across the Caco-2 cells following LPS (500 g/L) damage. (**A**) TEER was measured at each timepoint as the % of Transwell initial TEER measurement prior to the start of treatment. (**B**) FITC-dextran permeability across the Caco-2 cell monolayers measured following LPS exposure was unaffected by CFS at all concentrations. Statistical significance is annotated as * *p* < 0.05, and ** *p* ≤ 0.01.

In vitro Caco-2 cell studies were conducted to correlate in vivo outcomes in our HFD-fed rat study with the role of inulin metabolites as regulators of intestinal barrier integrity. Inulin_14_ was selected due to its significant impact on SCFA production demonstrated in the current study. We employed an in vitro fermentation system using commensal *Blautia* strains to generate cell-free supernatants (CFSs) from inulin_14_, simulating the metabolic by-products available in the gut environment post inulin fermentation. These CFSs, enriched with SCFAs and potentially other bioactive metabolites, were then applied to the previously developed LPS-damaged Caco-2 cell model. Eighteen and twenty-four hours post LPS removal from the Transwell, the CFSs (1 g/L) enhanced TEER by 16% and 13%, when compared the cell culture medium alone (Figure 10A). However, no significant differences in Caco-2 monolayer permeability were observed for the CFS and the HFD groups (Figure 10B). These findings suggest inulin_14_ may combat obesity by restoring gut barrier function, which is impaired by inflammation in metabolic diseases, as evidenced by the improved recovery of the Caco-2 cell monolayer.

## 4. Conclusions

Administering inulin with varying degrees of polymerization (DPn) of inulin to rats on a high-fat diet (HFD) revealed significant insights into its impact on metabolic health and the gut microbiome. Inulin_27_ demonstrated a notable reduction in HFD-induced weight gain and systemic glucose levels in rats, highlighting the potential of long-chain inulin to mitigate obesity and its metabolic biomarkers over just a 3-week study period. Distinct beneficial shifts in the gut microbiota were produced with inulin_7_ treatment, highlighting the preference of microbial species for relatively simple carbohydrate structures. Despite these promising outcomes, this study did not find any changes in IL-6 and TNF-α proinflammatory markers after the 3-week treatment period. The short treatment duration of the current study may not adequately capture the long-term impacts of inulin on inflammatory markers with sufficient statistical power. These findings call for more extensive studies to understand the role of inulin’s chemical structure in obesity management.

## Figures and Tables

**Table 1:** PERMANOVA analysis (Bray–Curtis and Jaccard indices).

Group 1	Group 2	Pseudo-f Statistic		*p*-Value	
Bray–Curtis	Jaccard	Bray–Curtis	Jaccard	Bray–Curtis	Jaccard
HFD	Inulin_7_	3.53714	2.53989	0.01732	0.01732
HFD	Inulin_14_	0.89221	0.83340	0.44589	0.53030
Inulin_7_	Inulin_14_	3.43465	2.63353	0.03030	0.02381
HFD	Inulin_27_	2.20498	1.85110	0.07576	0.07576
Inulin_7_	Inulin_27_	3.21460	2.84335	0.03247	0.01299
Inulin_14_	Inulin_27_	1.16436	0.94797	0.33333	0.44805

## Data Availability

The original contributions presented in the study are included in the article, further inquiries can be directed to the corresponding author.
