# Correction: Ariaee et al. The Degree of Inulin Polymerization Is Important for Short-Term Amelioration of High-Fat Diet (HFD)-Induced Metabolic Dysfunction and Gut Microbiota Dysbiosis in Rats. *Foods* 2024, *13*, 1039

**DOI:** 10.3390/foods13244030

**Published:** 2024-12-13

**Authors:** Amin Ariaee, Hannah R. Wardill, Anthony Wignall, Clive A. Prestidge, Paul Joyce

**Affiliations:** 1 UniSA Clinical and Health Sciences, University of South Australia, Adelaide, SA 5000, Australia; amin.ariaee@mymail.unisa.edu.au (A.A.); anthony.wignall@unisa.edu.au (A.W.); clive.prestidge@unisa.edu.au (C.A.P.); 2 School of Biomedicine, The University of Adelaide, Adelaide, SA 5000, Australia; hannah.wardill@adelaide.edu.au; 3 Supportive Oncology Research Group, Precision Cancer Medicine, South Australian Health and Medical Research Institute, Adelaide, SA 5000, Australia

## Error in Figure

In the original publication [1], there was a mistake in Figures 3B–D and 10B, as published. Several groups in these graphs had duplicated data due to an error in copying graph styles (maintaining symbols, colors, fonts etc.). The corrected, namely Figure 3B–D and Figure 10B, appear below. The authors state that the scientific conclusions are unaffected. This correction was approved by the Academic Editor. The original publication has also been updated.

## Figures and Tables

**Figure 3:**
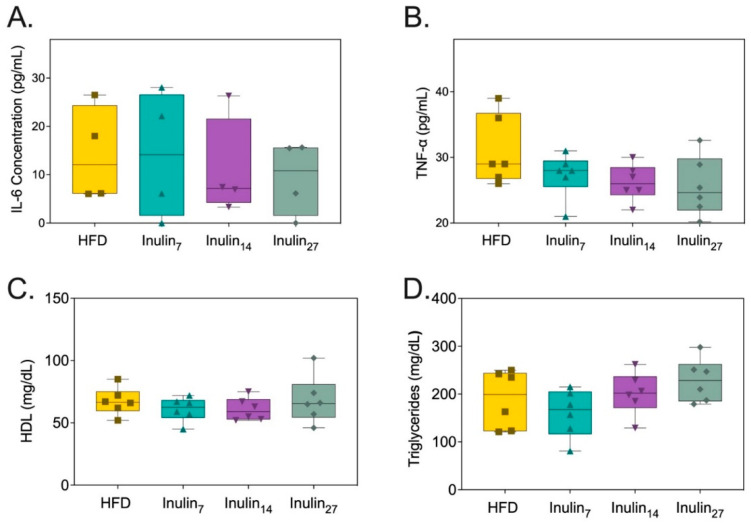
Metabolic markers of health measured from rats after 21 days of treatment. (**A**) Serum IL-6 concentration, (**B**) TNF-α concentration, (**C**) HDL concentration, and (**D**) triglyceride concentrations were not affected by inulin treatments.

**Figure 10:**
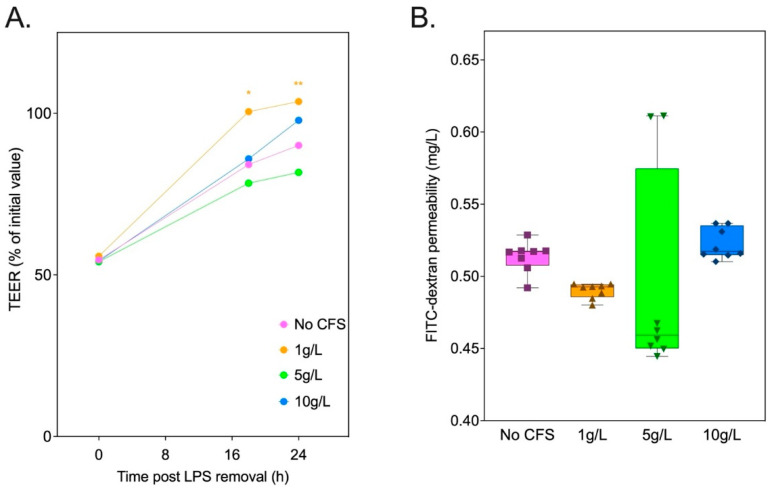
Cell-free supernatant (CFS) at 1 g/L of inulin_14_ fermented by commensal *Blautia* strains recovered TEER across the Caco-2 cells following LPS (500 g/L) damage. (**A**) TEER was measured at each timepoint as the % of Transwell initial TEER measurement prior to the start of treatment. (**B**) FITC-dextran permeability across the Caco-2 cell monolayers measured following LPS exposure was unaffected by CFS at all concentrations. Statistical significance is annotated as * *p* < 0.05, and ** *p* ≤ 0.01.
